# The Effects of IL-23/IL-18-Polarized Neutrophils on Renal Ischemia–Reperfusion Injury and Allogeneic-Skin-Graft Rejection in Mice

**DOI:** 10.3390/biomedicines11123148

**Published:** 2023-11-26

**Authors:** Changhong Wu, Jinglin Xu, Zhaoqi Zhang, Dong Wei, Yanan Xu, Yong Zhao

**Affiliations:** 1 State Key Laboratory of Membrane Biology, Institute of Zoology, Chinese Academy of Sciences, Beijing 100045, China; wuchanghong@ioz.ac.cn (C.W.); rsdyxjh0415@163.com (J.X.); xuyanan15@mails.ucas.ac.cn (Y.X.); 2 University of Chinese Academy of Sciences, Beijing 101408, China; 3 CAS Key Laboratory of Quantitative Engineering Biology, Shenzhen Institute of Synthetic Biology, Shenzhen Institute of Advanced Technology, Chinese Academy of Sciences, Shenzhen 518055, China; zzqsherry@126.com (Z.Z.); weidong94213@163.com (D.W.); 4 Faculty of Synthetic Biology, Shenzhen Institute of Advanced Technology, Shenzhen 518055, China

**Keywords:** neutrophils, ischemia–reperfusion injury, subsets, allogeneic transplantation, grafts, mice

## Abstract

Neutrophils display heterogeneity and plasticity with different subgroups and immune-regulatory functions under various surrounding conditions. Neutrophils induced by IL-23/IL-18 (referred to N(IL-23+IL-18) neutrophils) have a unique gene-expression profile, with highly expressing IL-17, MHC-II, and costimulatory molecules. The adoptive transfer of N(IL-23+IL-18) neutrophils significantly increased the pathogenesis in a renal ischemia–reperfusion injury mouse model. N(IL-23+IL-18) neutrophils directly and efficiently induced allogeneic T cell proliferation in vitro. N(IL-23+IL-18) neutrophils enhanced the syngeneic T cell response to allogeneic antigens in mixed-lymphocyte reaction assays. The adoptive transfer of the donor or host N(IL-23+IL-18) neutrophils significantly enhanced the antidonor antibody production in an allogeneic-skin-transplanted mouse model, accompanied by increased Tfh cells in the spleens. Therefore, the neutrophil subset induced by IL-23/IL-18 promotes tissue injury and antidonor humoral response in the allogeneic transplantation mouse model.

## 1. Introduction

Neutrophils are important innate immune cells and play a key role in human immune and inflammatory responses. As the first line of defense against bacterial and fungal infections, neutrophils eliminate invading pathogens through their phagocytic ability, as well as the production and release of reactive oxygen species, proteases, and extracellular traps [1,2,3]. Neutrophils not only exert cytotoxicity against invading pathogens, but also play a regulatory role in both innate and adaptive immunity [4,5]. Numerous studies have shown that neutrophils could upregulate the expression of MHC-II, CD80, and CD86 when stimulated by antigens or inflammatory factors. Thus, neutrophils acquire the function of antigen presentation [6,7,8,9]. In addition, neutrophils also play an important role in trained immunity [10]. Neutrophils are important for organ allograft rejection, and studies have shown that the recruitment of neutrophils to skin grafts can exacerbate skin rejection [11,12,13]. Ischemia–reperfusion injury of donor organs is an important factor in organ failure after transplantation, and neutrophil infiltration also plays an important role in renal ischemia–reperfusion injury (IRI) [14,15]. Importantly, neutrophils exhibit great plasticity and heterogeneity in different microenvironments. Therefore, the concept of neutrophil subpopulations has also been proposed. Based on density-gradient centrifugation, neutrophils can be divided into low-density neutrophils and high-density neutrophils [16,17]. Fridlender et al. reported the polarization state of proinflammatory and anti-inflammatory neutrophils [18]. They explained the tumor-promoting and antitumor effects of tumor-related neutrophils in this way, and referred to the neutrophil subunits of antitumor and tumor-promoting neutrophils as N1 and N2, respectively [18]. Our previous studies showed that IL-23-induced neutrophils express a unique gene-expression profile and participate in the pathological process of colitis [19]. IL-23/IL-18-polarized neutrophils have an impact on tumor- and collagen-induced arthritis in mice [20]. In contrast, IL-33-polarized neutrophils have unique cell-surface markers and gene profiles for cytokine/chemokine production, and play an important role in airway allergic inflammation [21]. These studies further confirm the diversity and plasticity of neutrophils.

IL-23 is a member of the IL-12 cytokine family, mainly secreted by activated dendritic cells and macrophages [22,23]. Endothelial cells, keratinocytes, and synovial cells can also release IL-23 [24]. IL-23 plays an important role in various inflammatory diseases, such as psoriasis, psoriatic arthritis, rheumatoid arthritis, and inflammatory bowel disease [25,26,27,28]. IL-23 can act on various immune cells in the immune response. In addition to its important role in the polarization of Th17 cells, IL-23 also plays a crucial role in γδT cells, natural killer T cells, and type 3 innate lymphoid cells [29,30]. The IL-23 receptor, as a receiver of IL-23 signals, is not only expressed in these cells, but also on innate immune cells, such as macrophages and dendritic cells [31,32]. IL-17 is an effector downstream of the IL-23 pathway, which is secreted by Th17 cells, γδT cells, congenital lymphocytes, natural killer cells, invariant NKT cells, neutrophils, and so on [33,34]. IL-17 plays an important role in protecting hosts from bacterial and other pathogens, and is also a major pathogenic cytokine in various autoimmune and inflammatory diseases [30], organ IRI [35,36,37], and allograft rejection [38,39,40,41].

In the present study, using myeloid-specific IL-23R-deficient mice and IL-17KO mice, we explored the roles of the IL-23- and IL-18-induced neutrophil subset in renal IRI, antigraft immune response, and the generation of antidonor antibodies in skin-transplanted mice. This study helps us understand the roles of different neutrophil subsets in allogeneic-organ-graft rejection and to explore new therapeutic strategies for donor organ ischemia–reperfusion injury or antibody-mediated organ-graft rejection.

## 2. Materials and Methods

### 2.1. Mice

Six-to-eight-week-old C57BL/6, BALB/c, and CD45.1^+^ mice were purchased from SPF Biotechnology Co., Ltd. (Beijing, China). BALB/c-GFP mice and C57BL/6-GFP mice were purchased from Biosaitu Bioengineering Co., Ltd. (Beijing, China). Six-to-eight-week-old IL-17A-KO mice were kindly provided by the Key Laboratory of Human Diseases Comparative Medicine, the Ministry of Public Health; Institute of Laboratory Animal Science, CAMS and PUMC (Beijing, China) [19]. Lyz-IL-23R-cKO mice with the C57BL/6 genetic background were obtained by crossing IL-23R flox/flox mice with Lyz2-Cre mice. All mice were maintained in a specific, pathogen-free facility, and were housed in microisolator cages containing sterilized feed, autoclaved bedding, and water. All experimental manipulations were undertaken in accordance with the Institutional Guidelines for the Care and Use of Laboratory Animals by the Institute of Zoology, CAS (Beijing, China).

### 2.2. Reagents

Anti-mCD11b-BV510, anti-mCD11b-BV395, anti-mLy6G-FITC, anti-mF4/80-PE, anti-mLy6G-PE, anti-mT-bet-PE, anti-mCD44-APC, anti-mCD45.2-APC, anti-mPD-1-BV605, anti-mCD8-PE-Cy5, anti-CXCR5, and Streptavidin-BV395 were purchased from BD (San Diego, CA, USA). Anti-mTCRβ-PE-Cy7, anti-mCD4-PE-Cy7, and anti-mH2Dd-FITC were purchased from Biolegend (San Diego, CA, USA). Fixable Viability Dye eFluor™ 780 and anti-mCD45-APC were purchased from eBioscience (San Diego, CA, USA). Anti-KIM 1 (catalog: ab78494) was purchased from Abcam (Cambridge, UK). The MojosortTM mouse neutral isolation kit was purchased from Miltenyi Biotec (Bergisch Gladbach, Germany). RmIL-18 and rmIL-23 were purchased from R&D Systems (Cambridge, UK).

### 2.3. Mouse Bone-Marrow Neutrophil Isolation

According to our previous protocol [42], mice were anesthetized and decapitated in an aseptic environment, soaked in 75% alcohol, and sucked dry with sterile absorbent paper. The femur, tibia, and iliac bones were separated, and the bone marrow cavities were then washed thoroughly with PBS to obtain the bone marrow cells. According to the instructions of the mojosortTM mouse neutral isolation kit introduction, non-neutrophil cells were combined with the antibodies, respectively, then discarded by the magnetic-activated cell-sorting (MASC) separation system. The purity of the neutrophils (CD11b^+^Ly6G^+^ cells) obtained with the MASC system was >95%, as detected by flow cytometry.

### 2.4. Polarization of the Neutrophils In Vitro

The purified mouse neutrophils were adjusted to 1 × 10^6^ cells/mL and added into 48-well plates. The cells were cultured with cytokines or inhibitors according to the following concentrations: rmIL-23 (10 ng/mL) and rmIL-18 (25 ng/mL).

### 2.5. Skin Transplantation

For immunization, tail skin grafts from the BALB/c mice were transplanted onto C57BL/6 mice, as described previously [43]. The hair was shaved with a clipper before the skin transfer. In order to be more easily distinguished, the direction of the BALB/c hair needed to be different from the direction of the C57BL/6 hair, reverse or upward, with three stitches on each side. For the rejection, the tail skin grafts from the BALB/c mice were transplanted onto the C57BL/6 mice, as described previously [43]. Seven days after the transplantation, skin graft photos were taken every one to two days with a digital camera until the graft was rejected.

### 2.6. Evaluation of Skin Necrosis

For the skin transplantation, erythema, edema, and hair loss were considered early signs of rejection, whereas ulceration, progressive shrinkage, and desquamation were considered to be the end-point of the rejection. Completely intact skin grafts had a score of 0, skin grafts with a necrotic area < 20% had a score of 1, skin grafts with a necrotic area of 20–40% had a score of 2, skin grafts with a necrotic area of 40–60% had a score of 3, skin grafts with a necrotic area of 60–80% had a score of 3, skin grafts with a necrotic area of 80–100% (but not dropped) had a score of 4, and skin grafts with a completely dropped area had a score of 5.

### 2.7. Isolation of Single Cells of Skin Tissue

According to our previous protocol [44], the skin was quickly cut into small pieces with a scalpel, then digested for 60 min (for the skin) or 40 min (for the heart) at 37 °C with 400 U/mL collagenase IV (Sigma-Aldrich, St. Louis, MO, USA), 10 mM HEPES, and 0.01% DNase I (MP Biomedicals, Irvine, CA, USA) in HBSS. Digested suspensions were passed through a nylon mesh. Then, the cells were collected after centrifugation at 300× *g* for 10 min.

### 2.8. IRI Mouse Model

Male mice (6–8 weeks old; weight 18–22 g) were anesthetized with 1% pentobarbital sodium on a heating pad at 37 °C. Rectal probes were used to detect and maintain the mouse temperature. After unclamping the noninvasive microvascular clamps, 5-0 silk thread was used to close the peritoneum and skin in sequence. An amount of 1 milliliter of physiological saline was injected through the intraperitoneal route to prevent dehydration. Mice were placed under a 12 h light/dark cycle, provided normal food at will, and sacrificed 24 h after the I/R. Kidney tissues were collected for histology and RNA extraction. The kidneys were fixed in 4% paraformaldehyde for H&E, which was carried out by Wuhan Servicebio Technology Co., Ltd. (Wuhan, China) for the assessment of renal damage.

### 2.9. Isolation of Single Cells in Kidneys

Twenty-four hours after the I/R, the mice were sacrificed and perfused with 0.9% NaCl solution. The kidneys were acquired and grinded. A single-cell suspension was digested at 37 °C for 30 min in 1640 medium containing 1 mg/mL type IV collagenase (Sigma-Aldrich, St. Louis, MO, USA) and 20 U/mL DNase I (Sigma-Aldrich, St. Louis, MO, USA).

### 2.10. Assessment of Renal Function

Serum creatinine was measured to assess the renal function and analyzed by Beijing Brightshines Technology Co., Ltd. (Beijing, China).

### 2.11. Allogeneic Mixed-Lymphocyte Reaction Assay

BALB/c spleen cells (stimulators) and C57BL/6 spleen cells (responders) were cultured in a ratio of 1:2 (1.5 × 10^5^:3 × 10^5^) in round-bottomed 96-well plates with RPMI 1640 medium containing 10% FBS. Stimulators were pretreated with mitomycin C, while responders were pretreated with CFSE. After coculturing for 5 days, the proliferation of responders was detected by flow cytometry.

### 2.12. Flow Cytometry

The cells from the harvested skin, kidneys, and spleens were washed once with PBS containing 0.1% NaN_3_ and 0.5% bovine serum albumin. Then, the cells were resuspended in PBS buffer and stained with antibodies. Flow cytometry data were acquired on an LSRFortessa™ X-20 instrument (BD Biosciences, San Jose, CA, USA) and analyzed with FlowJo V 10 software (Treestar, Ashland, OR, USA).

### 2.13. Quantitative PCR Analysis

Total RNA was isolated with TRIzol (Invitrogen, Carlsbad, CA, USA) and reverse transcribed with Superscript II (Qiagen, Dusseldorf, NRW, Germany) according to the manufacturer’s instructions. The cDNA served as template for the amplification of target genes and the housekeeping gene (HPRT) by real-time PCR. Target gene expression was calculated using the comparative method for relative quantification upon normalization to HPRT gene expression. The primers used in the present study are listed in Table 1.

### 2.14. RNA-Seq Analysis

Total RNA was isolated by Trizol, and the RNA purity was detected to ensure the concentration and integrity of the RNA samples to meet the quality requirements of transcriptome sequencing [45]. Next, we constructed the library, and the main process was as follows: (1) The eukaryotic mRNA was enriched with magnetic beads with Oligo-dT; (2) Random interruption of mRNA was achieved by adding fragment buffer; (3) The mRNA was used as a template to synthesize the first cDNA strand with six-base random hexamers, then the second cDNA strand was synthesized by adding buffer, dNTPs, RNase H, and DNA polymerase I. The cDNA was purified by ampere XP beads; (4) The purified double-stranded cDNA was then repaired, A-tailed, and connected to the sequencing adapter, and then AMPure XP beads were used for the fragment-size selection; (5) Finally, the cDNA library was obtained by PCR enrichment. Qubit 2.0 and Agilent 2100 were used to detect the concentration and insert size of the library, and the effective concentration of the library was accurately quantified using the Q-PCR method to ensure the quality of the library [20]. The NovaSeq 6000 was used for the high-throughput sequencing, and the sequencing read length was PE150. HISAT2 was used to align the reads to the mice genome (mm10), and StringTIe was used to construct transcripts independently for each cell [46]. DEseq2 was used to identify differentially expressed genes between each group [47]. KEGG pathway enrichment was performed by KOBAS 3.0 [48]. Protein and protein interaction networks were analyzed by String (https://string-db.org/, accessed on 27 October 2022) and visualized by Cytoscape [49].

### 2.15. Statistical Analysis

All data are presented as the mean ± SD. The Student unpaired *t*-test for the comparison of means was used to compare groups. A *p* < 0.05 was considered to be statistically significant.

## 3. Results

### 3.1. Alleviation of Renal IRI in Mice with Myeloid-Cell-Specific IL-23R Deficiency

**Figure 1:**
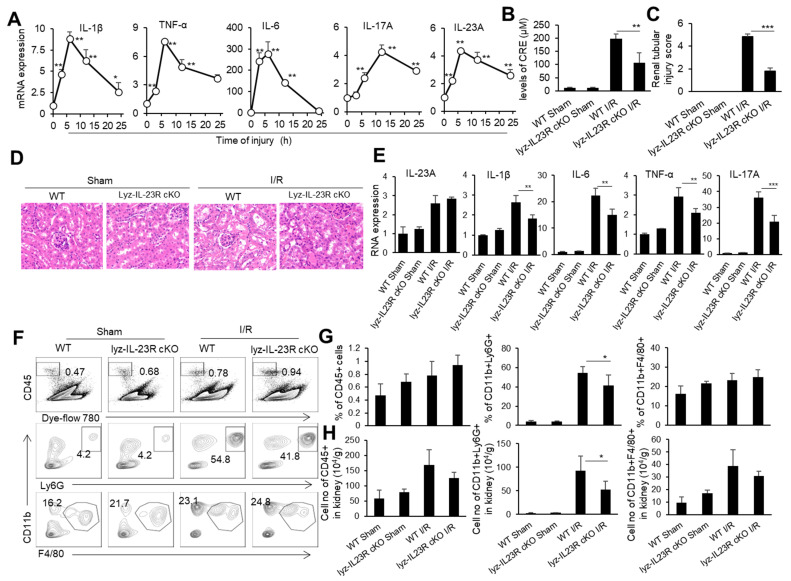
The cytokine mRNA expression, immune cell infiltration, and injury in the kidneys of WT and Lyz-IL-23R cKO mice after I/R. (**A**) Relative mRNA expression of IL-1β, TNF-α, IL-6, IL-17A, and IL-23A in the renal tissue of WT C57BL/6 mice at 0, 3, 6, 12, and 24 h after IR (*n* = 3). (**B**) Serum creatinine levels of WT and Lyz-IL-23R cKO mice in the sham operation group and I/R group at 24 h after I/R (*n* = 3). (**C**) Renal injury scores of the WT and Lyz-IL-23R cKO mice in the sham operation group and I/R group at 24 h after IR (*n* = 3). (**D**) Representative H&E-stained renal sections of WT and Lyz-IL-23R cKO mice in the sham operation group and I/R group at 24 h after I/R (*n* = 3). (magnification × 10000) (**E**) Relative mRNA expression of IL-1β, TNF-α, IL-6, IL-17A, and IL-23A in the renal tissue of WT and Lyz-IL-23R cKO mice in the sham operation group and I/R group at 24 h after I/R (*n* = 3). (**F**) Flow cytometry analysis of CD45^+^, CD45^+^CD11b^+^Ly6G^+^, and CD45^+^CD11b^+^F4/80^+^cells in the renal tissue of WT and Lyz-IL-23R cKO mice in the sham operation group and I/R group at 24 h after I/R (*n* = 3). (**G**) The proportion of CD45^+^, CD45^+^CD11b^+^Ly6G^+^, and CD45^+^CD11b^+^F4/80^+^ cells in the renal tissue of WT and Lyz-IL-23R cKO mice in the sham operation group and IR group at 24 h after I/R (*n* = 3). (**H**) The cell numbers of CD45^+^, CD45^+^CD11b^+^Ly6G^+^, and CD45^+^CD11b^+^F4/80^+^ cells in the renal tissue of WT and Lyz-IL-23R cKO mice in the sham operation group and I/R group at 24 h after I/R (*n* = 3). Data are means ± SD. * *p* < 0.05; ** *p* < 0.01; *** *p* < 0.001.

IRI is an important cause of organ-graft dysfunction, and the renal ischemia–reperfusion (I/R) model is a classic model for studying ischemia–reperfusion injury in solid organs. We subjected male C57BL/6 mice to 30 min of bilateral renal pedicle vascular occlusion at a constant temperature of 37 °C. We took kidney tissue from mice at 0, 3, 6, 12, and 24 h after the renal I/R and detected the mRNA levels of inflammatory factor TNF-α, IL-1β, IL-6, IL-23, and IL-17 in the kidneys through real-time PCR (Figure 1A). As expected, the levels of inflammatory cytokines TNF-α, IL-1β, and IL-6 in the kidneys rapidly rose to their peak within 6 h after the renal I/R, then slowly decreased within 24 h. Meanwhile, IL-23 and IL-17 in the renal tissue rapidly rose to its peak within 6 h of the renal I/R, while IL-17, the downstream cytokine of IL-23, rose to its peak within 12 h of the renal I/R, showing a delayed pattern compared to IL-23 (Figure 1A). It is known that neutrophils are important effector cells involved in renal IRI. IL-23 is the initiating factor of the IL-23/IL-17 pathway and plays an important role in the recruitment of neutrophils [50]. To investigate the effect of IL-23 on neutrophils during renal IRI, we constructed a renal IRI model in mice with myeloid-cell-specific IL-23R deficiency (Lyz-IL23R cKO). We set WT and Lyz-IL23R cKO mice with only an open abdominal cavity and free renal pedicle as the sham surgery control groups. We found that Lyz-IL23R cKO mice had lower blood creatinine levels than WT mice after the renal I/R (Figure 1B). Similarly, the HE and IHC staining of the renal pathological sections showed that Lyz-IL23R cKO mice had a decrease in renal tubular necrosis, with lower renal injury scores compared to WT mice after the renal I/R (Figure 1C,D). At the same time, the mRNA levels of inflammatory cytokines TNF-α, IL-1β, IL-6, and IL-17 in the renal tissue of Lyz-IL23R cKO mice were lower than those of WT mice after the renal I/R (Figure 1E). The knockout of IL-23R on myeloid cells did not affect the level of IL-23 in the mouse kidney tissue after the renal I/R, but did affect the expression level of IL-17. The proportion and cell numbers of neutrophils, and the cell numbers of CD45^+^ cells and macrophages, in the kidneys of Lyz-IL-23R cKO mice were less than those in WT mice after the renal I/R (Figure 1F,H). These results indicate that myeloid cells respond to IL-23 and contribute to IRI in mice.

### 3.2. Alleviation of Renal IRI in IL-17-Deficient Mice

**Figure 2:**
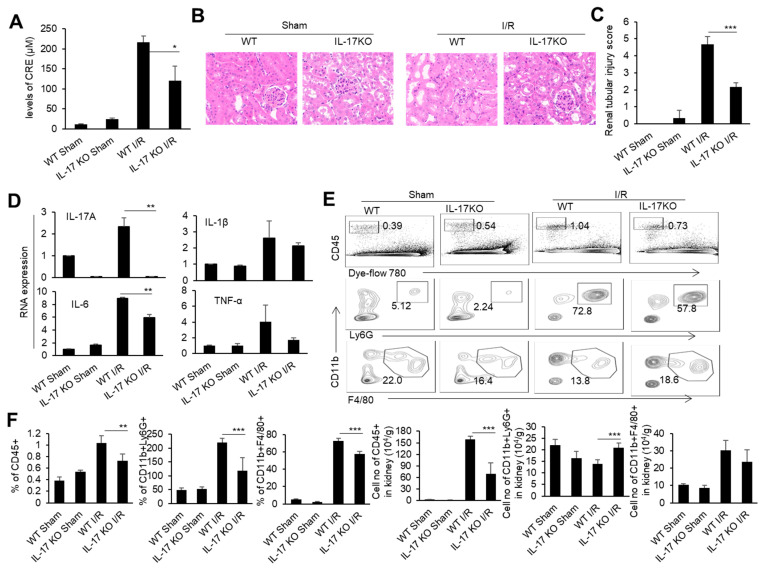
The cytokine mRNA expression, immune cell infiltration, and injury in the kidneys of the WT and IL-17 KO mice after I/R. (**A**) Serum creatinine levels of the IL-17KO mice in the sham operation group and I/R group at 24 h after I/R (*n* = 3). (**B**) Representative H&E-stained renal sections of the WT and IL-17KO mice in the sham operation group and I/R group at 24 h after I/R (*n* = 3). (magnification × 10000) (**C**) Renal injury scores of the WT and IL-17KO mice in the sham operation group and I/R group at 24 h after IR (*n* = 3). (**D**) Relative mRNA expression of IL-1β, TNF-α, IL-6, and IL-17A in the renal tissue of the WT and IL-17KO mice in the sham operation group and I/R group at 24 h after I/R (*n* = 3). (**E**) Flow cytometry analysis of CD45^+^, CD45^+^CD11b^+^Ly6G^+^, and CD45^+^CD11b^+^F4/80^+^ cells in the renal tissue of the WT and IL-17KO mice in the sham operation group and I/R group at 24 h after I/R (*n* = 3). (**F**) The proportion and cell numbers of CD45^+^, CD45^+^CD11b^+^Ly6G^+^, and CD45^+^CD11b^+^F4/80^+^ cells in the renal tissue of the WT and IL-17KO mice in the sham operation group and I/R group at 24 h after I/R (*n* = 3). Data are means ± SD. * *p* < 0.05; ** *p* < 0.01; *** *p* < 0.001.

To determine the role of IL-17 in renal IRI, we performed a renal IRI model in IL-17 KO mice, and detected various injury indicators at 24 h after the renal I/R. At the same time, WT mice and IL-17 KO mice with only an open abdominal cavity and free renal pedicle were used as the sham surgery control groups. The IL-17 KO mice had relatively low blood creatinine levels than the WT mice after the renal I/R (Figure 2A). Meanwhile, the IL-17 KO mice showed reduced renal tubular necrosis, with lower renal injury scores compared to the WT mice after the renal I/R, as indicated by the H&E and IHC staining of the renal pathological sections (Figure 2B,C). The levels of inflammatory factors TNF-α, IL-1β, and IL-6 in the renal tissue of the IL-17 KO mice were lower than those of the WT mice after the renal I/R, as detected by real-time PCR (Figure 2D). Similarly, the proportion and cell numbers of CD45^+^ cells and neutrophils, as well as the cell numbers of macrophages in the kidneys of the IL-17 KO mice, were less than those in the WT mice after the renal I/R (Figure 2E,F). Thus, IL-17 deficiency alleviates renal IRI in mice.

### 3.3. N(IL-23+IL-18) Neutrophils Promote Renal IRI Depending on IL-17

**Figure 3:**
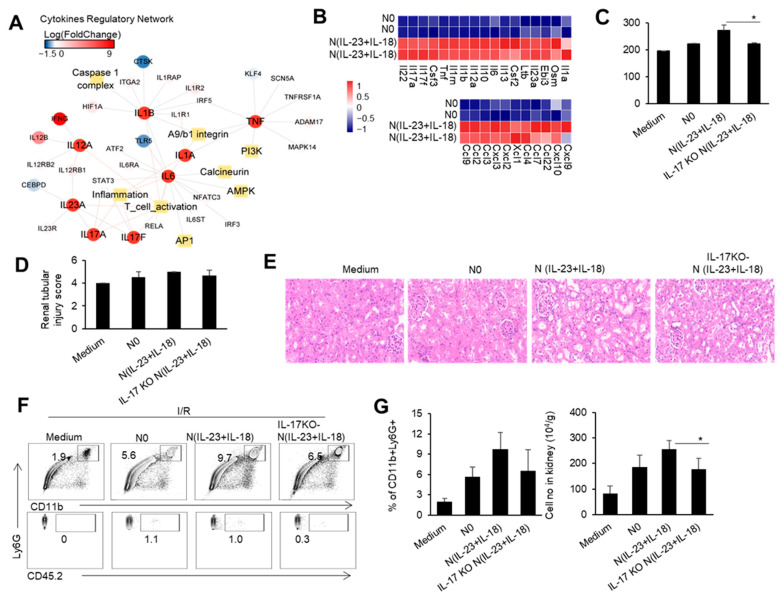
The cytokine and chemokine expression profile in N(IL-23+IL-18), and the effect of adoptively transferred WT and IL-17KO N(IL-23+IL-18) on the kidney IRI. (**A**) Cytokine regulatory network summary of N(IL-23+IL-18). (**B**) The heatmap of differentially expressed cytokines and cytokine receptors in N0 and N(IL-23+IL-18). (**C**) Serum creatinine levels in mice with adoptively transferred culture medium, N0, N(IL-23+IL-18), and IL-17KO N(IL-23+IL-18) neutrophils at 24 h after I/R (*n* = 3). (**D**) Renal tubular injury scores in mice with adoptively transferred culture medium, N0, N(IL-23+IL-18), and IL-17KO N(IL-23+IL-18) neutrophils at 24 h after I/R (*n* = 3). (**E**) Representative H&E-stained renal sections of mice with adoptively transferred culture medium, N0, N(IL-23+IL-18), and IL-17KO N(IL-23+IL-18) neutrophils at 24 h after I/R (*n* = 3). (magnification × 10000) (**F**) Flow cytometry analysis of CD11b^+^Ly6G^+^ and CD45.2^+^CD11b^+^Ly6G^+^ cells in the renal tissue of mice with adoptively transferred medium, N0, N(IL-23+IL-18), and IL-17KO N(IL-23+IL-18) neutrophils at 24 h after I/R (*n* = 3). (**G**) The proportion and cell numbers of CD11b^+^Ly6G^+^ and CD45.2^+^CD11b^+^Ly6G^+^ cells in the renal tissue of mice with adoptively transferred medium, N0, N(IL-23+IL-18), and IL-17KO N(IL-23+IL-18) neutrophils at 24 h after I/R (*n* = 3). Data are means ± SD. * *p* < 0.05.

Our previous studies showed that IL-23 combined with IL-18 induced IL-17 expression in neutrophils [20]. Therefore, we speculate that the increase in IL-23 after the renal I/R may directly affect the expression of IL-17 in neutrophils, which may enhance IRI pathogenesis. We used IL-23/IL-18 to induce the neutrophils and obtained the neutrophil subgroup N(IL-23+IL-18). By sequencing and analyzing the transcriptome of the N(IL-23+IL-18) cells, we found that the regulatory network of IL-23 and IL-17 cytokines in the N(IL-23+IL-18) cells was significantly upregulated compared with uninduced N0 neutrophils. Meanwhile, the regulatory networks related to inflammatory factors, such as TNF-α, IL-1α, IL-1β, and IL-6, were also significantly upregulated (Figure 3A). In addition to the high expression of cytokines and inflammatory factors mentioned above, N(IL-23+IL-18) also exhibited significant overexpression of certain chemokines, such as CCL2, CCL3, CXCL9, and so on (Figure 3B). To investigate the potential role of the neutrophil N(IL-23+IL-18) subset and neutrophil-produced IL-17 in the mouse renal IRI process, we performed mouse renal IRI studies with an adoptive transfer of different subsets of WT and IL-17KO neutrophils 4hr before the I/R. Mice with an adoptive transfer of neutrophil N(IL-23+IL-18) had significantly higher blood creatinine levels compared with mice in other groups after the renal I/R, while mice with adoptively transferred IL-17KO N(IL-23+IL-18) neutrophils had lower blood creatinine levels compared to mice with WT N(IL-23+IL-18) neutrophils (Figure 3C). Similarly, the HE and IHC staining of the pathological sections of the kidneys showed that the renal injury of mice with N(IL-23+IL-18) neutrophils was more severe than that of other groups, and the expression of the KIM-1 protein was more pronounced in these mice. However, the mice with IL-17KO N(IL-23+IL-18) neutrophils had less renal injury and significantly lower KIM-1 protein expression compared with mice with WT N(IL-23+IL-18) neutrophils (Figure 3D,E). Furthermore, the proportion and cell numbers of total CD11b^+^Ly6G^+^ cells in the kidney tissues of mice with adoptively transferred N(IL-23+IL-18) neutrophils were higher than those of other groups. The proportion and cell numbers of total CD11b^+^Ly6G^+^ cells in mice with IL-17KO N(IL-23+IL-18) neutrophils were similar to those in the group with WT N0 neutrophils (Figure 3F,G). Thus, neutrophil-produced IL-17 likely contributes to the damage process during IRI.

**Figure 4:**
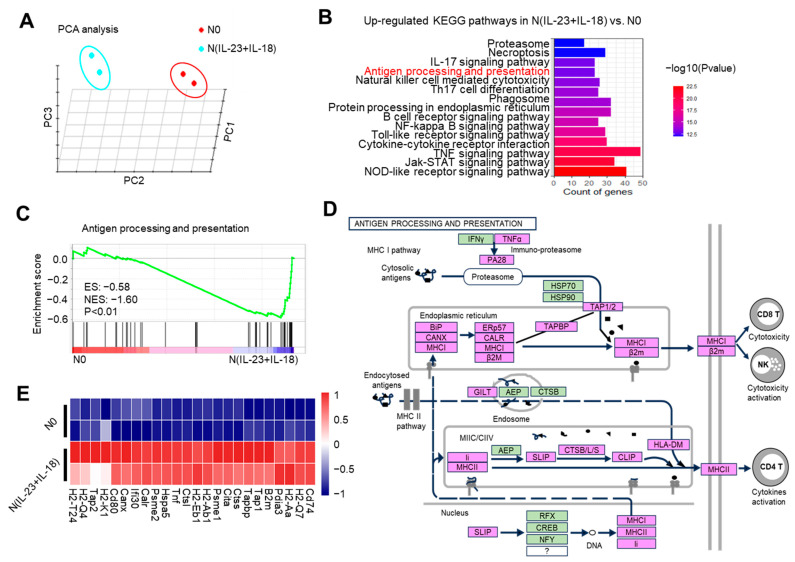
The potential enhanced antigen processing and presentation of N(IL-23+IL-18) as detected by RNA-seq. (**A**) Principal component analysis of neutrophils treated with none and IL-23/IL-18. All expressed genes are used for the analysis. Each group contains two parallel samples labeled in the same color. (**B**) Column chart of the KEGG pathway enrichment analysis. (**C**) Analysis of the GSEA enrichment related to antigen processing and presentation. (**D**) Genes and signaling pathways associated with a high expression of antigen processing and presentation in N(IL-23+IL-18) neutrophils. (**E**) Heatmap of the significantly changed MHC-II and costimulatory molecules.

To observe the gene transcription characteristics of N(IL-23+IL-18) neutrophils, we conducted RNA-seq assays of N0 and N(IL-23+IL-18) neutrophils. The distance of N0 and N(IL-23+IL-18) neutrophils were analyzed using principal component analysis (PCA) in three dimensions (Figure 4A). PCA clustering reflects a significant difference between N(IL-23+IL-18) and N0 neutrophils, which are two different cell subpopulations. KEGG pathway enrichment analysis showed that, after the combined stimulation of IL-23/IL-18, the cell-activation-related pathways of the neutrophils were significantly upregulated. In addition, the antigen-processing and presentation pathways were also significantly upregulated (Figure 4B). We conducted gene set enrichment analysis (GSEA) on the genes related to antigen processing and presentation, and found that these genes were significantly enriched in N(IL-23+IL-18) neutrophils (Figure 4C). We plotted a possible pathway diagram for antigen-processing and presentation-related high-expression genes to transmit antigen signals in N(IL-23+IL-18) neutrophils (Figure 4D). From the thermogram of the differentially expressed genes between the N0 and N(IL-23+IL-18) neutrophils, it can be clearly seen that the expression of MHC-II, costimulatory molecules, and surface molecules related to T and B cell activation in N(IL-23+IL-18) neutrophils are obviously increased (Figure 4E).

**Figure 5:**
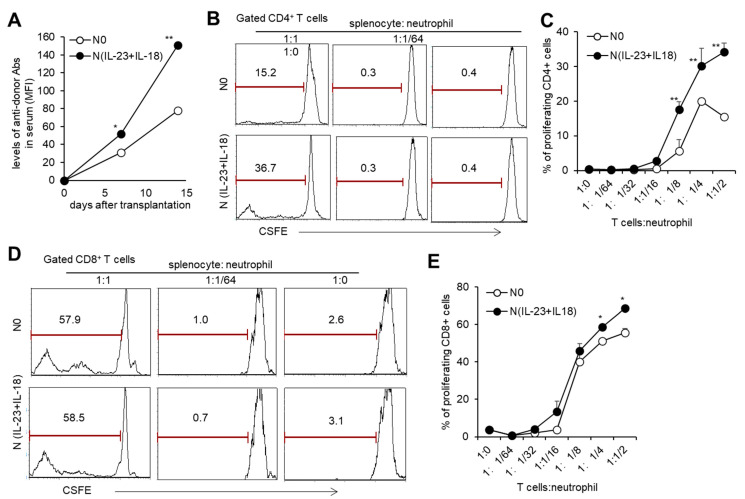
T cell proliferation stimulated by allogeneic N(IL-23+IL-18) neutrophils in vitro. (**A**) The levels of antidonor antibodies in the sera of C57BL/6 mice received allogeneic N0 and N(IL-23+IL-18) neutrophils on day 0, 7, and 14 after adoptive transfer (*n* = 3). (**B**) Flow cytometric analysis of the proliferation of CD4^+^ T cells stimulated by allogeneic N0 and N(IL-23+IL-18) neutrophils in vitro (*n* = 3). (**C**) Statistical analysis of CD4^+^ T cell proliferation stimulated by allogeneic N0 and N(IL-23+IL-18) neutrophils, respectively (*n* = 3). (**D**) Flow cytometric analysis of the proliferation of CD8^+^ T cells stimulated by allogeneic N0 and N(IL-23+IL-18) neutrophils. (**E**) Statistical summary of CD8^+^ T cell proliferation stimulated by allogeneic N0 and N(IL-23+IL-18) neutrophils, respectively (*n* = 3). Data are means ± SD. * *p* < 0.05; ** *p* < 0.01.

With the sequencing analysis of N(IL-23+IL-18) neutrophils showing that they might have an enhanced antigen-presenting function, we studied the effect of allogeneic N(IL-23+IL-18) neutrophils on T cell activation in vitro. When the ratio of T cells to neutrophils was greater than 1:1/8, both allogeneic N0 and N (IL-23+IL-18) neutrophils could promote the proliferation of T cells, and N(IL-23+IL-18) neutrophils had a stronger ability to promote T cell proliferation compared with N0 neutrophils (Figure 5B–E), indicating that allogeneic N(IL-23+IL-18) neutrophils can directly promote the proliferation of allogeneic T cells. To see the immunogenicity of N(IL-23+IL-18) neutrophils in vivo, we injected B6 N(IL-23+IL-18) neutrophils into allogeneic BALB/c mice and collected blood from each group of mice to obtain mouse sera for the detection of the levels of antidonor antibodies on the 0th, 7th, and 14th days. We found that N0 and N(IL-23+IL-18) neutrophils induced a certain level of antidonor antibody production, and N(IL-23+IL-18) neutrophils induced significantly higher levels of antidonor antibodies than N0 neutrophils (Figure 5A). These results indicate that donor N(IL-23+IL-18) neutrophils promote a stronger humoral immune response in vivo.

### 3.4. The Effect of Donor N(IL-23+IL-18) Neutrophils on Allogeneic-Skin-Graft Rejection

**Figure 6:**
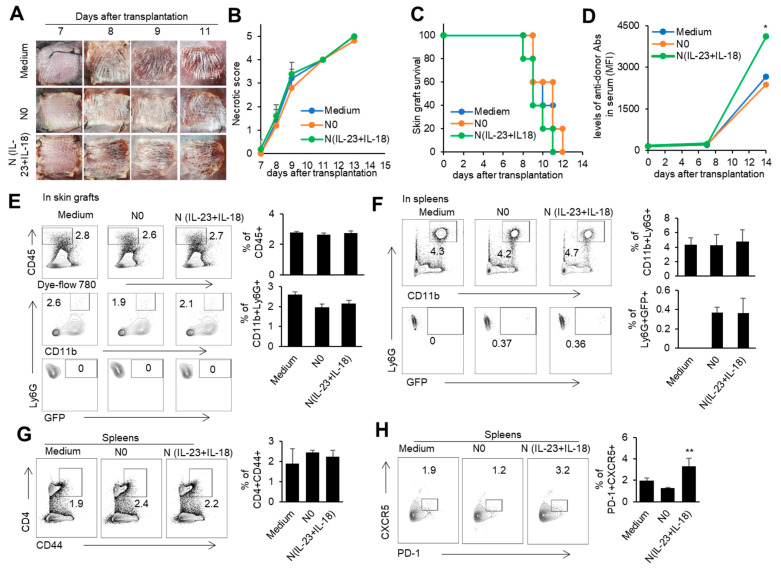
The effects of adoptively transferred donor N(IL-23+IL-18) neutrophils on the allogeneic donor skin graft survival and antidonor IgG production. (**A**) Photos of BALB/c skin grafts in C57BL/6 mice with adoptively transferred allogeneic donor N0 and N(IL-23+IL-18) neutrophils (*n* = 5). (**B**) Necrotic score of BALB/c skin grafts at different time points (*n* = 5). (**C**) Survival rate of BALB/c skin grafts in C57BL/6 mice with adoptively transferred donor N0 and N(IL-23+IL-18) neutrophils (*n* = 5). (**D**) The levels of antidonor IgG antibodies in the sera of C57BL/6 mice with adoptively transferred donor N0 and N(IL-23+IL-18) neutrophils on day 0, day 7, and day 14 after skin transplantation (*n* = 3–4). (**E**) Flow cytometric analysis of the infiltration and proportion of allogeneic donor GFP^+^ neutrophils in BALB/c skin grafts of C57BL/6 recipient mice with adoptively transferred donor GFP^+^ N0 and N(IL-23+IL-18) neutrophils on the 9th day after skin transplantation (*n* = 4). (**F**) Flow cytometric analysis of the infiltration and proportion of GFP^+^ neutrophils in the spleens of C57BL/6 recipient mice with the adoptively transferred medium, donor GFP^+^ N0, and N(IL-23+IL-18) neutrophils on the 9th day after skin transplantation (*n* = 5). (**G**) Flow cytometric analysis of CD4^+^CD44^+^ cells and their proportion in the spleen of C57BL/6 recipient mice with the adoptively transferred donor N0 and N(IL-23+IL-18) neutrophils on the 9th day after BALB/c skin transplantation (*n* = 5). (**H**) Flow cytometric analysis of Tfh cells and their proportion in the spleens of C57BL/6 recipient mice with adoptively transferred donor N0 and N(IL-23+IL-18) neutrophils on the 9th day after BALB/c skin transplantation (*n* = 3). Data are means ± SD. * *p* < 0.05; ** *p* < 0.01.

In order to investigate the role of donor N(IL-23+IL-18) neutrophils in transplantation, we used a mouse model of skin transplantation with an i.v. injection of donor N0 or N(IL-23+IL-18) neutrophils, as described in the Methods. Allogeneic donor B6 skin grafts in BALB/c recipients of all groups were rejected within 12 days after grafting (Figure 6A–C). Moreover, the level of antidonor IgG in the sera of mice with adoptively transferred donor N(IL-23+IL-18) neutrophils was significantly higher than that of the other two groups (Figure 6D). Although the adoptive transfer of donor N (IL-23+IL-18) neutrophils did not significantly change the rejection time of the skin grafts, the increase in antidonor antibody production indicates that donor N(IL-23+IL-18) neutrophils have a promoting effect on the host humoral immune response. According to the above phenomenon, we want to further explore whether donor N(IL-23+IL-18) neutrophils directly participate in the inflammatory reaction by infiltrating into skin grafts, or migrate to the spleen as antigen-presenting cells to present antigens, and to activate the adaptive immune response in skin-rejection models. In order to facilitate the detection of neutrophil infiltration in mice, we used BABL/c mice expressing the GFP fluorescent protein to prepare donor N0 and N(IL-23+IL-18) neutrophils, and adoptively transferred these neutrophils to allogeneic C57BL/6 recipient mice to detect the infiltration of the adoptively transferred neutrophils on the 9th day after transplantation (Figure 6E,F). We found that, at the time of rejection, the adoptively transferred GFP^+^ neutrophils were not detected in the skin graft, but a certain proportion of GFP^+^ neutrophil infiltration was detected in the spleens of the mice, indicating that donor N(IL-23+IL-18) neutrophils can migrate to the recipient spleen to exert immune effects. Considering that N(IL-23+IL-18) neutrophils express high levels of MHC-II molecules and costimulatory molecules, and promote antidonor antibody production, we speculate that N(IL-23+IL-18) neutrophils may promote the polarization of Tfh subsets [51]. The analysis of mouse spleen cells showed that donor N(IL-23+IL-18) neutrophils resulted in an increase in Tfh levels compared with N0 neutrophils. Similarly, the levels of the Tfh1-related transcription factor T-bet in the spleens of the mice also increased after the injection of N(IL-23+IL-18) neutrophils. (Figure 6G,H). Thus, donor N(IL-23+IL-18) neutrophils have the ability to promote antidonor antibody production in mice.

### 3.5. The Effect of Recipient N(IL-23+IL-18) Neutrophils on Allogeneic-Skin-Graft Rejection

**Figure 7:**
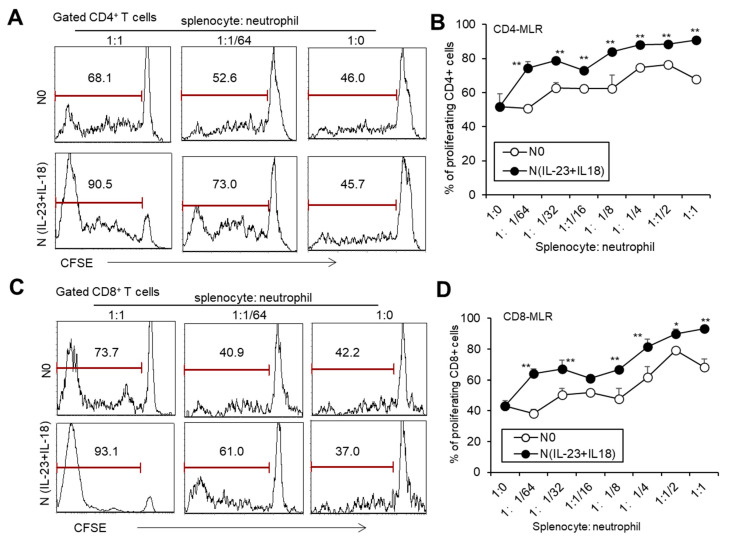
The effects of syngeneic N(IL-23+IL-18) neutrophils on T cell proliferation stimulated by allogeneic antigens in MLR assays. (**A**) Flow cytometry was used to detect the proliferation of CD4^+^ T cells stimulated by allogeneic antigens in the presence of syngeneic N0 and N(IL-23+IL-18) neutrophils, respectively (*n* = 3). (**B**) Statistics of CD4^+^ T cell proliferation stimulated by allogeneic antigens in the presence of syngeneic N0 and N(IL-23+IL-18) neutrophils in vitro (*n* = 3). (**C**) Flow cytometry was used to detect the proliferation of CD8^+^ T cells stimulated by allogeneic antigens in the presence of syngeneic N0 and N(IL-23+IL-18) neutrophils, respectively (*n* = 3). (**D**) Summary of the proliferation of CD8^+^ T cells stimulated by allogeneic antigens in the presence of syngeneic N0 and N(IL-23+IL-18) neutrophils, respectively (*n* = 3). Data are means ± SD. * *p* < 0.05; ** *p* < 0.01.

**Figure 8:**
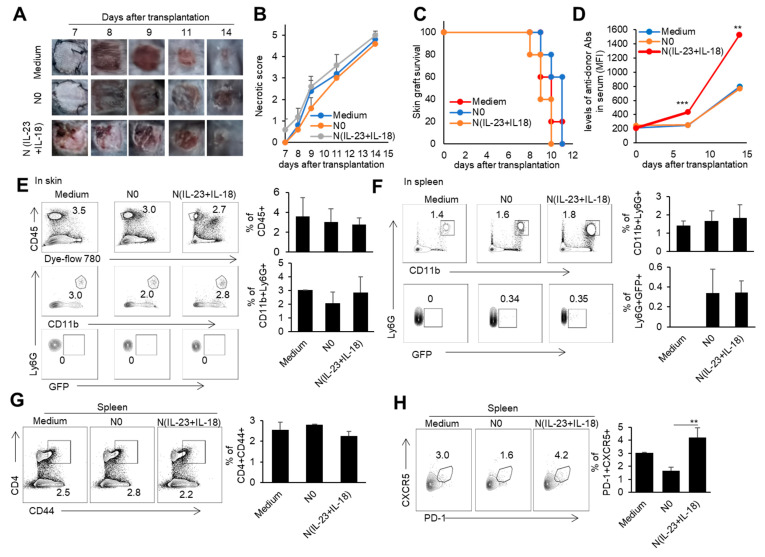
The effects of the adoptively transferred host N(IL-23+IL-18) neutrophils on the allogeneic donor skin graft survival and antidonor IgG production. (**A**) Photos of BALB/c skin grafts in C57BL/6 mice with adoptively transferred syngeneic host N0 and N(IL-23+IL-18) neutrophils (*n* = 5). (**B**) Necrotic score of BALB/c skin grafts at different time points (*n* = 5). (**C**) Survival rate of BALB/c skin grafts in C57BL/6 mice with the adoptively transferred host N0 and N(IL-23+IL-18) neutrophils (*n* = 5). (**D**) The levels of antidonor IgG antibodies in the sera of C57BL/6 mice with the adoptively transferred host N0 and N(IL-23+IL-18) neutrophils on day 0, day 7, and day 14 after skin transplantation (*n* = 3–4). (**E**) Flow cytometric analysis of the infiltration and proportion of host GFP^+^ neutrophils in BALB/c skin grafts in C57BL/6 mice with the adoptively transferred donor GFP^+^ N0 and N(IL-23+IL-18) neutrophils on the 9th day after skin transplantation (*n* = 3). (**F**) Flow cytometric analysis of the infiltration and proportion of GFP^+^ neutrophils in the spleen of C57BL/6 recipient mice with the adoptively transferred medium, donor GFP^+^ N0, and N(IL-23+IL-18) neutrophils on the 9th day after skin transplantation (*n* = 3). (**G**) Flow cytometric analysis of CD4^+^CD44^+^ cells and their proportion in the spleens of C57BL/6 recipient mice with the adoptively transferred host N0 and N(IL-23+IL-18) neutrophils on the 9th day after BALB/c skin transplantation (*n* = 3). (**H**) Flow cytometric analysis of Tfh cells and their proportion in the spleens of C57BL/6 recipient mice with the adoptively transferred host N0 and N(IL-23+IL-18) neutrophils on the 9th day after BALB/c skin transplantation (*n* = 3). Data are means ± SD. ** *p* < 0.01; *** *p* < 0.001.

To investigate whether recipient N(IL-23+IL-18) neutrophils play a role in allogeneic graft rejection, we employed a mixed-lymphatic-culture system with the addition of recipient N0 and N(IL-23+IL-18) neutrophils into the classic one-way mixed-lymphocyte reaction assays in vitro, in which irradiated allogeneic BALB/c splenocytes were used as allogeneic-stimulating antigens and B6 splenocytes as responder cells. We found that recipient N(IL-23+IL-18) neutrophils significantly enhanced the proliferation of recipient T cells in a mixed-lymphatic-culture system compared with N0 neutrophils (Figure 7A–D). To validate the effect of recipient N(IL-23+IL-18) neutrophils on allogeneic skin rejection, we used BALB/c tail skin as a donor graft and transplanted it to B6 recipient mice with the injection of C57BL/6 N(IL-23+IL-18) neutrophils. The adoptive transfer of recipient N(IL-23+IL-18) neutrophils did not significantly alter the time course of skin-graft rejection (Figure 8A–C). However, the antidonor IgG levels in the sera of mice with the adoptive transfer of recipient N(IL-23+IL-18) neutrophils were significantly higher than those of the other two groups (Figure 8D). Based on the above phenomena, we wanted to further explore how recipient N(IL-23+IL-18) neutrophils promote an immune response in skin-rejection models. To facilitate the neutrophil infiltration in recipient mice, we used C57BL/6 mice expressing the GFP fluorescent protein to prepare the recipient N0 and N(IL-23+IL-18) neutrophils, and detected the infiltration of the adoptively transferred syngeneic neutrophils on the 9th day after transplantation (Figure 8E,F). We were unable to detect the transferred GFP^+^ neutrophils in the skin grafts, but a certain proportion of GFP^+^ cell infiltration was detected in the spleens of the mice, indicating that recipient N(IL-23+IL-18) neutrophils can migrate to the recipient spleens and play a role. Meanwhile, the overall level of Tfh cells in the mouse spleens was significantly increased after the adoptively transferred recipient N(IL-23+IL-18) neutrophils compared to other experimental groups (Figure 8G,H). This result can explain the increase in antidonor antibody levels.

## 4. Discussion

Neutrophils are the main reactive cells during renal IRI, and they will infiltrate the tissue in a short period of time after reperfusion. Neutrophils can cause injury to kidney tissue by releasing proteases, myeloperoxidase, cytokines, and reactive oxygen species [52]. A study on renal IRI showed that IL-17 activation induced by the IL-23–IL-17 axis could promote the recruitment and migration of neutrophils, thereby accelerating renal IRI [53]. However, whether IL-23 can directly act on neutrophils during renal IRI has not been discussed. In the present study, we found that mice with a myeloid-specific IL-23R deficiency showed a lower blood creatinine level and renal-injury degree after the renal I/R decreased compared with WT mice. In addition, neutrophil infiltration and the levels of various inflammatory factors, including IL-17, in the kidney tissue of IL-23R cKO mice was also lower than that of the WT mice. After the renal I/R, the IL-17 KO mice showed a decrease in blood creatinine levels, reduced renal injury, and decreased levels of inflammatory factors in renal tissue compared with the WT mice, which is consistent with previous reports [54]. With the deepening research on neutrophils, the concept of neutrophil subpopulations has become increasingly important for researchers, and has been widely discussed and summarized [55]. Our previous studies have shown that the Th17-like neutrophil subpopulation that specifically secretes IL-17 after induction by IL-23 or the IL-23/IL-18 combination has the characteristics of promoting tumor growth and inflammation [19,20]. In the present study, the adoptive transfer of N(IL-23+IL-18) neutrophils enhanced the renal IRI response, but the adoptive transfer of IL-17 KO N(IL-23+IL-18) neutrophils failed to do so. These results indicate that IL-17 may be involved in the N(IL-23+IL-18) neutrophil-mediated IRI process.

Neutrophils not only have universal anti-infection and proinflammatory effects, but also have a wide range of other functions. For example, neutrophils express MHC-II molecules and costimulatory molecules to mediate T cell responses [56], and neutrophil subpopulations express CD40L, BAFF, APRIL, and IL-21 to activate B cells in the splenic marginal zone [57,58,59]. After the sequencing analysis of neutrophil N(IL-23+IL-18), we found that, in addition to overexpressing MHC-II molecules and costimulatory molecules, N(IL-23+IL-18) neutrophils also significantly increased the expression level of genes related to antigen-processing and extraction pathways. Neutrophils are important markers of transplant injury and play an important role in transplantation [60]. Neutrophils can produce reactive oxygen species to exacerbate tissue injury after transplantation [61], exacerbating acute cell rejection by stimulating dendritic cells [62] or T cells [63]. In addition, the increase in neutrophils in grafts is also related to antibody-mediated transplant rejection [64,65]. We cocultured N(IL-23+IL-18) neutrophils with sorted allogeneic T cells in vitro and found that N(IL-23+IL-18) neutrophils could promote the proliferation of allogeneic T cells. Next, we conducted in vivo experiments. We adoptively transferred donor N(IL-23+IL-18) neutrophils in a mouse allogeneic-skin-transplantation model and attempted to investigate the possible impact of donor N(IL-23+IL-18) neutrophils present in donor organs on transplant rejection. In this model, we found that donor N(IL-23+IL-18) neutrophils could not significantly alter the rejection time of skin grafts, but could infiltrate into the mouse spleen and promote the production of antidonor antibodies. The secretion of high-affinity antibodies was completed by plasma cells [66]. The ability of B cells to select and differentiate into plasma cell depended on the signal from Tfh cells in GCs [67]. Consistently, we found that donor N(IL-23+IL-18) neutrophils could promote the polarization of Tfh in the spleens of recipient mice.

On the other hand, we investigated the effects of recipient N(IL-23+IL-18) neutrophils on allograft rejection and antidonor humoral response. Recipient N(IL-23+IL-18) neutrophils could also promote T cell proliferation in a classical mixed-lymphocyte reaction assay. The adoptive transfer of recipient N(IL-23+IL-18) neutrophils did not significantly change the allo-skin-graft rejection time, but they could infiltrate into the mouse spleen and promote the production of antidonor antibodies and splenic Tfh polarization. Thus, both donor and host N(IL-23+IL-18) neutrophils could promote antidonor antibody production in a mouse transplantation model.

In summary, our current results showed that the combined IL-23/IL-18 induction of neutrophils promoted renal IRI through IL-17. N(IL-23+IL-18) neutrophils have the ability to promote allogeneic T cell proliferation. Both donor and host N(IL-23+IL-18) neutrophils could promote antidonor antibody production in a mouse transplantation model, likely by enhancing Tfh polarization. Our present research highlights the involvement of donor and host IL-23 and IL-18 costimulated neutrophil subpopulations in graft-tissue damage and the generation of antidonor antibodies during allograft transplantation; blocking this subpopulation may prevent allograft rejection.

## Figures and Tables

**Table 1:** Primers used for qRT–PCR analysis.

Genes		Primer Sequence (5′–3′)
HPRT	Forward primer:	AGTACAGCCCCAAAATGGTTAAG
	Reverse primer:	CTTAGGCTTTGTATTTGGCTTTTC
IL-1β	Forward primer:	TGGGAAACAACAGTGGTCAGG
	Reverse primer:	CCATCAGAGGCAAGGAGGAA
IL-6	Forward primer:	AACCGCTATGAAGTTCCTCTC
	Reverse primer:	AATTAAGCCTCCGACTTGTGAA
IL-17A	Forward primer:	CTCAGACTACCTCAACCGTTCC
	Reverse primer:	ATGTGGTGGTCCAGCTTTCC
IL-23A	Forward primer:	CTGAGAAGCAGGGAACAAGATG
	Reverse primer:	GAAGATGTCAGAGTCAAGCAGGTG
Tnf-α	Forward primer:	GAGTGACAAGCCTGTAGCC
	Reverse primer:	CTCCTGGTATGAGATAGCAAA

## Data Availability

Data are contained within the article.

## Abbreviations

CFSE	Carboxyfluorescein succinimidyl amino ester
GC	Germinal center
GFP	Green fluorescent protein
KEGG	Kyoto Encyclopedia of Genes and Genomes
KIM-1	Kidney injury molecule-1
I/R	Ischemia–reperfusion
IRI	Ischemia–reperfusion injury
PCA	Principal component analysis
PCR	Polymerase chain reaction
PD-L1	Programmed death-ligand 1
RNA-seq	Messenger ribonucleic acid sequencing
Tfh	Follicular helper T cell
